# Dynamic expression of Notch-dependent neurogenic markers in the chick embryonic nervous system

**DOI:** 10.3389/fnana.2014.00158

**Published:** 2014-12-18

**Authors:** Leslie Ratié, Michelle Ware, Hélène Jagline, Véronique David, Valérie Dupé

**Affiliations:** ^1^CNRS UMR6290, Faculté de Médecine, Institut de Génétique et Développement de Rennes, Université de Rennes 1Rennes, France; ^2^Laboratoire de Génétique Moléculaire, CHU PontchaillouRennes Cedex, France

**Keywords:** hypothalamus, early axon scaffold, spinal cord, neuronal differentiation, sensory nervous system, olfactory epithelium, cranial ganglia

## Abstract

The establishment of a functional nervous system requires a highly orchestrated process of neural proliferation and differentiation. The evolutionary conserved Notch signaling pathway is a key regulator of this process, regulating basic helix-loop-helix (bHLH) transcriptional repressors and proneural genes. However, little is known about downstream Notch targets and subsequently genes required for neuronal specification. In this report, the expression pattern of Transgelin 3 (*Tagln3*), Chromogranin A (*Chga*) and Contactin 2 (*Cntn2*) was described in detail during early chick embryogenesis. Expression of these genes was largely restricted to the nervous system including the early axon scaffold populations, cranial ganglia and spinal motor neurons. Their temporal and spatial expression were compared with the neuronal markers Nescient Helix-Loop-Helix 1 (*Nhlh1*), Stathmin 2 (*Stmn2*) and HuC/D. We show that *Tagln3* is an early marker for post-mitotic neurons whereas *Chga* and *Cntn2* are expressed in mature neurons. We demonstrate that inhibition of Notch signaling during spinal cord neurogenesis enhances expression of these markers. This data demonstrates that *Tagln3, Chga* and *Cntn2* represent strong new candidates to contribute to the sequential progression of vertebrate neurogenesis.

## Introduction

The central and peripheral nervous system of vertebrates arises from tissue with various embryological origins. During early development neurons develop from the neuroepithelium in specific regions of the brain and spinal cord within the central nervous system. The peripheral nervous system arises from placodal ectoderm as well as from the migratory neural crest cells (NCCs). Despite their different origins, differentiation of neurons throughout the embryo is similarly regulated by multiple genes, which are co-ordinately involved (Cordes, 2001).

Essentially, Notch signaling is the common pathway known to play a key role in the timing of neural progenitor cell differentiation in most, if not all, metazoans (reviewed in Dyer, 2003; Ishibashi, 2004; Louvi and Artavanis-Tsakonas, 2006). Upon activation of Notch signaling, the Notch intracellular domain (NICD)-recombination signal sequence binding protein K (RBPJ) complex induces expression of genes such as transcriptional repressors from the *Hes* and *Hey* families. It is well established that this Notch canonical pathway regulates many biological events, like neurogenesis, by repressing expression of target proneural basic helix-loop-helix (bHLH) genes such as *Ascl1* and *Neurog1/2*, thereby inhibiting neuronal differentiation. Such a mechanism is already implicated in the differentiation of most neuronal tissues throughout the nervous system (Bertrand et al., 2002). Proneural genes are required to induce neuronal differentiation. They are responsible for the formation of a bHLH transcription factor network that regulates the balance of different subtypes of neurons generated from neural progenitor cells. Transcription factors such as Neurogenic Differentiation 1 (NEUROD1) or Nescient Helix-Loop-Helix 1 (NHLH1) subsequently elicit the expression of terminal differentiation markers (Bertrand et al., 2002). While many components of this Notch/proneural network have been identified, little is known about the kinetics of downstream molecular events that lead to specific neural differentiation and specification.

In a previous study, we took advantage of the pharmacological γ-secretase inhibitor N-[3.5-difluorophenacetyl-L-alanyl)]-S-phenylglycine t-butyl ester (DAPT) to inactivate Notch signaling during early neurogenesis in the chick embryo to identify new genes involved in the differentiation cascade leading to hypothalamic neurons differentiation (Ratié et al., 2013). The use of microarray analysis has allowed us to characterize new targets of this Notch/proneural network such as Transgelin 3 (*Tagln3/NP22/NP25*), Chromogranin A (*Chga*) and Contactin 2 (*Cntn2/Tag-1*). TAGLN3, is an actin-binding protein involved in cytoskeletal organization (Mori et al., 2004), while CHGA is a member of the Granin family of neuroendocrine secretory proteins, located in secretory vesicles of neurons (Taupenot et al., 2003) and CNTN2 is a surface glycoprotein that has been described in early motor and commissural neurons in the developing neural tube (Stoeckli and Landmesser, 1995). Less is known about the function and expression domains of these genes in vertebrates, in particular with relation to other established neural differentiation factors. Considering neural differentiation occurs simultaneously with migration, the anatomical position of the expression domains of these genes relative to other genes whose functions are known, will contribute to defining the functional position of these particular genes in the neural differentiation hierarchy.

This study reports the expression of *Tagln3, Chga* and *Cntn2* genes during early chick development. Expression patterns were characterized in detail relative to *Nhlh1* and Stathmin 2 (*Stmn2/SCG10*), known markers during early neurogenesis as well as the pan-neuronal protein HuC/D. This data demonstrates that these genes are new specific markers for detecting neural differentiation as early as Hamburger and Hamilton stage (HH) 10.

## Methods

### Embryo preparation

Fertilized chicken (*Gallus gallus*) eggs were obtained from E.A.R.L. Les Bruyères (France). Animal experimentation protocols conformed to the European Union guidelines (RL2010/63/EU) and ethical approval was not required. Eggs were incubated in a humidified incubator at 38°C until the required developmental stages. The embryonic stages were determined according to Hamburger and Hamilton (1951). Embryos were fixed in 4% PFA/PBS at 4°C overnight, rinsed and processed for whole-mount RNA *in situ* hybridization.

Roller cultures and DAPT treatment were performed on HH9 chick embryos. Embryos were dissected and transferred into bijou tubes, in which they were cultured overnight either in Dimethyl sulfoxide (DMSO) or 40 μM DAPT-supplemented medium (Dupé et al., 2011). With such conditions, the size of the embryos was similar between DAPT-treated and control embryos with no obvious morphological defects. The embryos were used for *in situ* hybridization and processed for vibratome sections.

Sectioning microtome and vibratome: for paraffin sections, embryonic tissue was dehydrated through an ethanol series, embedded in paraffin, sectioned at 7 μm, deparaffinized and processed for *in situ* hybridization. For vibratome sectioning, whole embryos were placed into 4% low melting agarose blocks after *in situ* hybridization. Transversal sections (50–100 μm) were cut using a VT1200 microtome (Leica Biosytems).

### *In situ* hybridization and immunohistochemistry

mRNA riboprobes were obtained by PCR amplification using primers designed from NCBI sequences of each gene. cDNA was cloned into the pCRII TOPO vector (Invitrogen) and linearized DNA was transcribed to generate digoxigenin labeled sense and antisense riboprobes. These riboprobes were used for whole mount *in situ* hybridization on chick embryos as previously described (Ratié et al., 2013).

After *in situ* hybridization, embryos were dehydrated with methanol overnight to improve permeability of the antibody for immunohistochemistry (Lumsden and Keynes, 1989). Anti-HuC/D mouse (1:500; molecular probes; A21271) primary antibody was used and detected with a peroxidase-conjugated rabbit-anti-mouse secondary antibody (1:2000; Jackson ImmunoResearch; 315-035-045).

### Preparation and imaging of embryos

Stained embryos were photographed in 80% glycerol on a LEICA MZ16 APO and DM4000 microscope using LEICA LAS Software. Images were processed using Photoshop CS6 (Adobe Systems).

## Results

### Kinetic expression study of Nhlh1, Tagln3, Chga, Cntn2 and Stmn2 during early neurogenesis in the chick embryo

The developmental expression patterns of *Tagln3, Chga* and *Cntn2* were analyzed by whole-mount *in situ* hybridization between HH8 and HH22. The expression was compared with *Nhlh1*, an early pan-neuronal marker and *Stmn2*, a well-known marker of terminally differentiated neurons (Groves et al., 1995; Murdoch et al., 1999).

**Figure 1:**
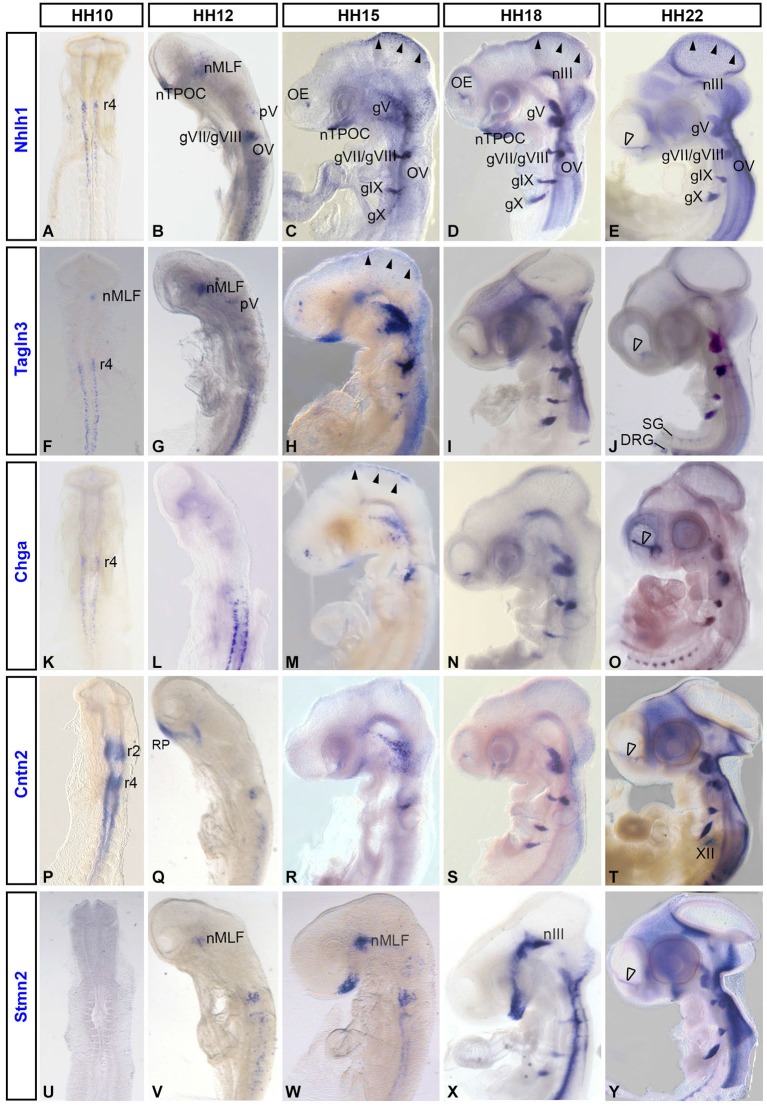
**Expression of the neuronal markers *Nhlh1, Tagln3, Chga, Cntn2* and *Stmn2* in the developing chick nervous system detected by whole-mount *in situ* hybridization**. *Nhlh1* **(A–E)**, *Tagln3* **(F–J)**, *Chga* **(K–O)**, *Cntn2* **(P–T)** and *Stmn2* **(U–Y)** mRNA expressions were reported from HH10 to HH22 on dorsal (HH10) and lateral views of whole-mount chick embryos. Roman numerals indicated expression in the cranial ganglia of the corresponding nerves. Arrowheads in **(C), (D), (E)**, indicated expression in the roof of mesencephalon. Unfilled arrowheads indicated expression in neurons migrating along the olfactory nerve towards the telencephalon. DRG: dorsal root ganglia; gV: trigeminal ganglion; gVII/VIII: facial and vestibulocochlear ganglia; gIX: petrosal ganglion; gX: nodose ganglion; nIII: nucleus of the oculomotor nerve; nMLF: nucleus of the medial longitudinal fascicle; nTPOC: nucleus of the postoptic commissure; OE: olfactory epithelium; OV: otic vesicle; r2, r4, r5: rhombomeres 2, 4 and 5; RP: Rathke’s pouch. SG: sympathetic ganglia; XII: hypoglossal nerve.

*Nhlh1, Tagln3, Chga, Cntn2* and *Stmn2* expression was not detected before HH8 (data not shown). At HH10, *Nhlh1, Tagln3 and Chga* were similarly expressed in scattered cells along the lateral neural tube, caudal to rhombomere 4 (r4; Figures 1A,F,K). *Cntn2* expression was more widespread throughout the medial-lateral axis at HH10 and strongly present along the dorsoventral axis of rhombomere 2 (r2) and r4 (Figure 1P). *Stmn2* expression was first detected from HH12 in cells along the neural axis, later than the other markers, reflecting its status as a late pan-neuronal marker (Groves et al., 1995).

Within populations of the early axon scaffold in the rostral brain, *Tagln3* was expressed within neurons of the nucleus of the medial longitudinal fascicle (nMLF) rostral to the diencephalic-mesencephalic boundary and first detected as early as HH10, earlier than *Nhlh1* and *Stmn2* (Figures 1A,F,U). *Nhlh1 and Stmn2* were first detected in the nMLF at HH12 (Figures 1B,V). *Nhlh1* was expressed in the nucleus of the tract of the postoptic commissure (nTPOC) within the developing hypothalamus as early as HH12 and the expression domain expanded during development within this region (Figures 1B–E) *Chga* was yet to be expressed in the brain (Figure 1L). From HH15, all markers except *Cntn2* were expressed in the developing hypothalamus within the nTPOC (Figures 1C,H,M,R,W). While* Stmn2* and *Tagln3* expression was maintained until HH22, *Chga* expression had disappeared in the developing hypothalamus by HH18 (Figures 1H–J,M–O,W–Y). At HH15, transient expression of *Nhlh1, Tagln3*, and *Chga* was present in the roof of mesencephalon that corresponded to the nucleus of descending tract of the mesencephalic nucleus of the trigeminal (nmesV; Figures 1C,H,M; arrowheads). At HH18, *Nhlh1, Tagln3 and Stmn2* expression appeared in a short stripe running along the ventral edge of the rostral mesencephalon that corresponded to the nucleus of the oculomotor nerve (nIII; Figures 1D,I,X).

At HH12, adjacent to the developing hypothalamus, *Cntn2* expression was detected in Rathke’s pouch, a small lateral stripe distal to the optic vesicles. This expression domain was not associated with the neuroectoderm when the neural tube was dissected (Figure 1Q; data not shown). At HH12, *Nhlh1* and *Tagln3* were the first markers to be detected in a few cells of the trigeminal placode (opV) that will contribute to the future ganglion (Figures 1B,G). By HH15, *Nhlh1* and *Tagln3* were expressed in all the cranial ganglia (Figures 1C,H) while *Chga* and *Cntn2* were only expressed in gV and gVI/VIII (Figures 1M,R) and *Stmn2* was only expressed in the gVI (Figure 1W). From HH18, all the markers were expressed in all the cranial ganglia including *Stmn2* (Figures 1D,I,N,S,X). Analysis at HH22 showed an interesting new expression domain for *Cntn2* in the hypoglossal nerve (XII; Figure 1T).

Interestingly, these neuronal markers shared specific expression during olfactory sensory neuron differentiation. By HH15, *Nhlh1, Tagln3* and *Chga* were detected in the olfactory epithelium (OE; Figures 1C,H,M). *Cntn2* was first detected at HH18 in the olfactory area while *Stmn2* was not expressed until between HH18 and HH22 (Figures 1S,X,Y). At HH22, all these markers were present in the migratory cells along the olfactory nerve bundle (Figures 1E,J,O,T,Y; unfilled arrowhead).

Comparison of the expression patterns of these neuronal differentiation markers during early neurogenesis indicated that these genes were globally expressed in most of the neuronal structures throughout the chick embryo between HH10 and HH22. A significant difference was the initiation of gene expression as these genes did not appear at the same time. Furthermore, in these tissues *Nhlh1, Tagln3, Chga* and *Cntn2* were largely expressed before the late differentiation marker *Stmn2*.

### Novel markers assume differential expression during different phases of neuronal differentiation within the developing chick brain

**Figure 2:**
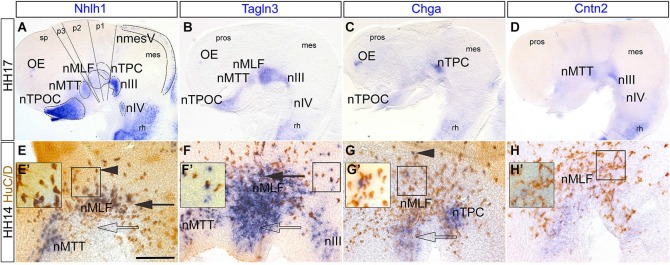
**Expression of *Nhlh1, Tagln3, Chga* and *Cntn2* in the rostral chick brain detected by whole-mount *in situ* hybridization. (A–D)** HH17. Lateral view of flat-mounted rostral brain. *Nhlh1* **(A)**, *Tagln3* **(B)**, *Chga* **(C)** and Cntn2 **(D)** expression was present within various populations of the early axon scaffold and somatic motor neurons in the rostral chick brain (delimited by dotted lines in **(A)**, mostly along the ventral midline of the brain. **(E–H)** HH14. High magnification images of the medial longitudinal fascicle (MLF) in the caudal prosencephalon. Double labeling of markers with the pan-neural antibody HuC/D. Scale bar: 100 μm. **(E)** *Nhlh1* was expressed by the nucleus of mamillo-tegmental tract (nMTT) and central population of the MLF (filled arrow). **(F)** *Tagln3* was expressed in the nMTT, central (filled arrow) and ventral populations (unfilled arrow) of the MLF as well as the nucleus of oculomotor nerve (nIII). **(G)** *Chga* expression overlapped within the ventral region of the MLF (unfilled arrow) and the nucleus of the tract of the posterior commissure (nTPC). There were some HuC/D positive neurons within the dorsal MLF population that expressed *Chga* (arrowhead). **(H)** *Cntn2* was not expressed by neurons but expression was present along the ventral midline. **(E’–H’)** Higher magnification images of boxes indicated in **(E–H)**. This highlighted HuC/D positive cells that were either positive for the marker or negative for the marker. In the case of *Tagln3* **(F’)** some cells were HuC/D negative. mes: mesencephalon; nIV: nucleus of the trochlear nerve; nmesV: mesencephalic trigeminal nucleus; nTPOC: nucleus of the tract of the postoptic commissure; OP: olfactory placode; pros: prosencephalon; p1–3: prosomeres 1 to 3; rh: rhombencephalon.

To determine the expression of these markers inside the rostral neural tube, the brain was dissected to reveal the prosomeres (Figure 2A), allowing the identification of many populations of the early axon scaffold (Puelles et al., 1987; Puelles and Rubenstein, 2003). Here, *Nhlh1, Tagln3, Chga* and *Cntn2* expression was analyzed in the chick rostral brain at HH17 (Figures 2A–D), when the principal tracts of the chick early axon scaffold have become established (Ware and Schubert, 2011). At HH17, these populations included the nucleus of the MLF (nMLF), the first axon tract to form in the rostral brain within prosomeres 1 and 2. The hypothalamus contained two set of neurons, those that belonged to the tract of the postoptic commissure (TPOC), in the rostral end of the basal hypothalamus and those of the mamillotegmental tract (MTT). The nucleus of the tract of the posterior commissure (nTPC) differentiated in prosomere 1 at the diencephalic-mesencephalic boundary. Somatic motor neurons in the ventral mesencephalon (oculomotor; III) and at the mesencephalic-rhombencephalic boundary (trochlear; IV) also differentiated during establishment of the early axon scaffold (Chilton and Guthrie, 2004).

The overview analysis of the dissected brain revealed that *Nhlh1* and *Tagln3* were expressed in the nTPOC, nMTT, nMLF nIII and nIV whereas expression was absent in the nTPC (Table 1 and Figures 2A,B). *Chga* was intensely expressed in the nTPC and weak expression was found at the level of the nTPOC, coinciding with downregulation by HH18 (Figures 1N, 2C). *Cntn2* expression was more specific to the nIII, while expression was also present in the nMTT and nIV (Figure 2D). Expression within the caudal diencephalon was analyzed further to explore expression within the first neurons to differentiate in the rostral brain forming the nMLF. These neurons were organized into three separate populations that occupy different positions located centrally, dorsally and ventrally (Ware and Schubert, 2011). Thus, these specific populations of MLF neurons have been used to compare co-expression of the neuronal markers with the pan-neuronal marker, HuC/D, to confirm the expression of *Nhlh1, Talgn3, Chga* and *Cntn2* within mature neurons. Chick brains were analyzed at HH14 just as the three populations of MLF neurons became established. HuC/D immunostaining in the neuronal cell bodies was much denser in the central MLF population than in the dorsal and ventral MLF populations at this stage (Figures 2E–H). Double labeling with HuC/D revealed expression of *Nhlh1* within neurons of the central MLF population (Figure 2E, arrow) and some scattered neurons of the more dorsal MLF population (Figures 2E,E’, arrowhead). *Tagln3* was densely expressed within the central and ventral MLF populations (Figure 2F, arrow and unfilled arrow). Interestingly, *Nhlhl1* expression was restricted to most neural cell bodies expressing HuC/D but not all (Figures 2E,E’). *Tagln3* appeared to be expressed by both cells that were HuC/D positive post-mitotic neurons but also cells that did not express HuC/D yet, although these cells were most likely destined to become neurons (Figures 2F,F’). This was evident in the ventral nMLF population and also in the nIII where the first post-mitotic neurons appeared at HH15. Previous studies have shown that these regions will eventually become neuronal (Puelles et al., 1987; Ware and Schubert, 2011). As *Tagln3* was not expressed by all HuC/D positive neurons, this suggested *Talgn3* expression was downregulated once the neuron has matured.* Chga* expression overlapped within the ventral nMLF population but did not appear to be co-expressed by the HuC/D positive neurons in this region (Figure 2G, unfilled arrow). There was some co-expression in the dorsal population of MLF neurons (Figures 2G,G’, arrowhead). There was also expression in cells of the nTPC that had yet to become post-mitotic (Figure 2G). Remarkably, a specific marker for the population of neurons of the nTPC is yet to be described therefore *Chga* could be a useful marker. *Cntn2* was expressed in the ventral midline but nor the MLF neurons (Figures 2H,H’).

**Table 1:** **Expression of the markers in neuronal populations of the chick rostral brain at HH17**.

*Nhlh1* (Figure 2A)	*Tagln3* (Figure 2B)	*Chga* (Figure 2C)	*Cntn2* (Figure 2D)
nTPOC	nTPOC	nTPOC
nMTT	nMTT		nMTT
nMLF	nMLF
		nTPC
nIII	nIII		nIII
nIV	nIV		nIV

**Figure 3:**
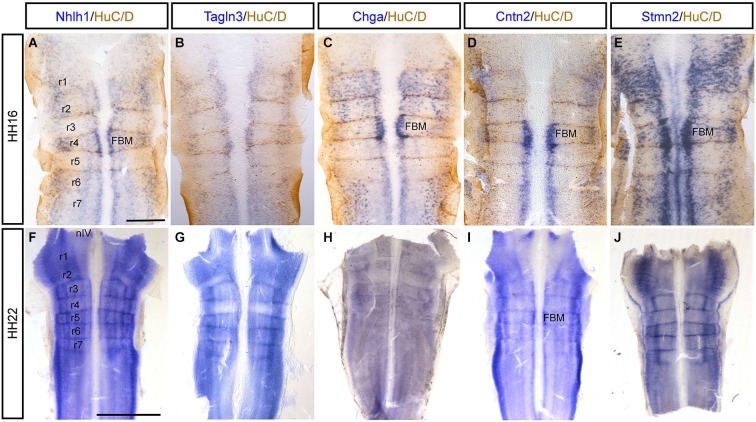
***Tagln3, Chga* and *Cntn2* expression pattern in the developing chick rhombencephalon in comparison to *Nhlh1* and *Stmn2*. (A–E)** A dorsal view of the rhombencephalon, flat-mounted at HH16. Double labeled with HuC/D antibody and mRNA probes. Rhombomeres were clearly visible at this time (r1–r7). Cell bodies of the motor nerves were located at different mediolateral positions within the rhombencephalon. **(F–J)** At HH22, as the number of neurons increased, the expression of these markers became more widespread and diffuse. Scale bars: 100 μm. nVI: nucleus of the abducens nerve. FBM, facial branchiomotor neurons. FP: floor plate.

To investigate the expression of these neuronal markers in the rhombencephalon, flat-mounted preparations at HH16 were examined after rhombomere boundaries were established. *In situ* hybridization combined with immunostaining for HuC/D showed that *Stmn2* was expressed in all the cell bodies of the neurons whereas *Nhlh1, Tagln3, Chga* and *Cntn2* expression was more limited (Figures 3A–E). For all markers, a spotted expression was detected in the dorsal part of the neural tube in the region corresponding to the developing somatic motor neurons (Guthrie, 2007). Expression of *Nhlh1, Chga, Cntn2* and *Stmn2* was detected ventrally where branchiomotor and visceral motor neurons differentiate adjacent to the floor plate (Figures 3A–E). The expression was stronger in the basal plate of the r4 (Figures 3A–E) corresponding to the formation of the facial branchiomotor neurons (Garel et al., 2000). By HH22, expression of these markers appeared diffuse throughout much of the rhombencephalon (Figures 3F–J). *Tagln3* expression appeared to be downregulated in r2 and r4 (Figure 3G). *Cntn2* was the only marker still strongly expressed in the neurons of facial branchiomotor neurons (Figure 3I).

### Cranial sensory neuron differentiation proceeds by sequential expression of Tagln3, Chga and Cntn2

The role of these neuronal markers during differentiation was examined further in the cranial sensory ganglia with respect to post-mitotic neurons using double labeling of the markers with HuC/D. This analysis was performed at HH18 after the peak of placodal neuroblasts migration (Freter et al., 2013).

**Figure 4:**
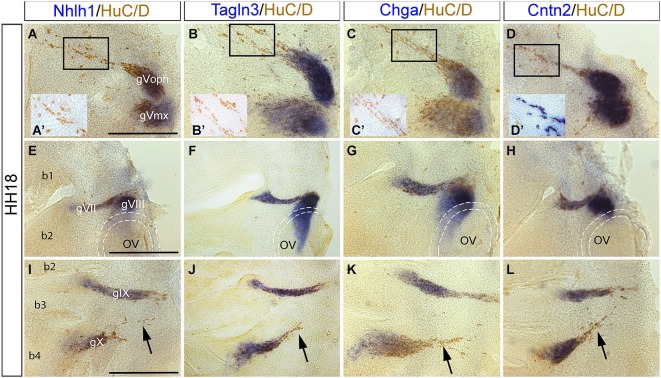
**Expression of *Nhlh1, Tagln3, Chga* and *Cntn2* in the cranial ganglia by whole-mount *in situ* hybridization and anti HuC/D immunostaining at HH18. (A–D)** *Nhlh1, Chga, Tagln3* and *Cntn2* were expressed in neurons of the trigeminal ganglia (gV) but expression was not exclusive to the post-mitotic neurons. **(A’–D’)** Small insert panels show enlargements of the migrating neurons associated to the ophthalmic lobe of the trigeminal ganglion (gVoph). *Nhlh1, Chga* and *Tagln3* were not co-expressed in these migrating neurons, whereas *Cntn2* was expressed **(D’). (E–H)** *Nhlh1, Chga, Tagln3* and *Cntn2* were expressed in neurons of facial (gVII) and vestibulocochlear (gVIII) ganglia. In the vestibulocochlear ganglia, *Chga* and *Tagln3* expression expanded more ventrally than *Nhlh1* and *Cntn2* in cells that were not HuC/D positive. Dotted lines delimited the otic vesicles. **(I–L)** *Nhlh1, Chga, Tagln3* and *Cntn2* were expressed in neurons of glossopharyngeal (gIX) and nodose ganglia (gX). Only *Cntn2* was expressed in the dorsal part of the nodose ganglia corresponding to migrating neurons. Arrows indicate the migrating neurons from the nodose ganglion (arrows). Scale bars: 100 μm. b1, b2, b3 and b4: branchial arches; OV: otic vesicles.

Flat-mounted preparations of the gV revealed expression of these markers in the ophthalmic (oph) and maxillomandibular (mx) lobes of the trigeminal ganglion (Figures 4A–D). Similar to the analysis of the nMLF, expression of *Nhlh1, Tagln3* and *Chga* only partially overlapped with HuC/D (Figures 4A’–C’). However, *Cntn2* appeared to be expressed in all post-mitotic neurons including mature migratory neurons from the ophthalmic branch of the gV (Figure 4D’).

In flat-mounted preparations of the otic area overlapping expression of the four markers occurred within HuC/D positive neurons in the gVII and gVIII ganglia (Figures 4E–H). *Nhlh1, Tagln3* and *Chga* were also expressed in HuC/D negative cells behind the otic vesicle (Figures 4E–G). These cells were neuronal progenitors that migrated away from the otic vesicle epithelium to form sensory neurons of the gVIII (Fekete and Wu, 2002).

*Nhlh1, Chga, Tagln3* and *Cntn2* were similarly expressed in the gIX and gX ganglia (Figures 4I–L). These two ganglia were produced by migration of sensory neurons from epibranchial placodes (Steventon et al., 2014). At the level of the gX, differential expression of these markers was observed in different populations of HuC/D positive post-mitotic cells. In the distal portion cells that were migrating towards the rhombencephalon (Figures 4I–L; arrows), did not express *Nhlh1, Chga* and *Tagln3* but expressed *Cntn2*. Interestingly, in the cranial ganglia, only *Cntn2* was expressed in the migratory neurons that were the most differentiated neurons at this stage (Figures 4A–D,I–L).

### Nhlh1, Tagln3, Chga and Cntn2 were expressed by olfactory placodal cells and migratory neurons

**Figure 5:**
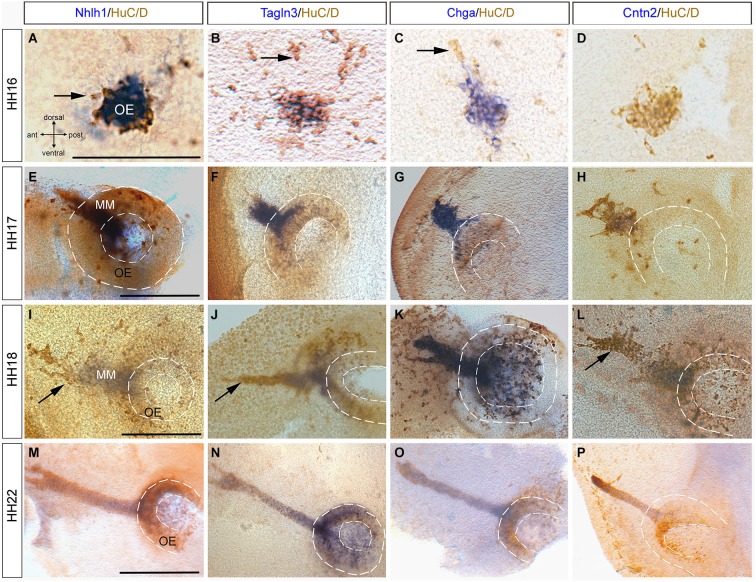
**Expression of *Nhlh1, Tagln3, Chga* and *Cntn2* in the developing olfactory placode by whole-mount *in situ* hybridization and anti-HuC/D immunostaining from HH16 to HH22. (A–D)** At HH16 *Nhlh1, Tagln3* and *Chga* were expressed in neurons in the olfactory placodal region, whereas *Cntn2* was not. Arrows indicated neuronal cells that were HuC/D positive only. **(E–H)** At HH17, *Chga* and *Tagln3* had a similar expression in the developing olfactory epithelium (OE) which extended to the migrating cells **(F,G). (H)** *Cntn2* began to be expressed in some neurons of the developing olfactory epithelium. **(I–L)** At HH18 *Nhlh1, Chga, Tagln3* and *Cntn2* were co-expressed in neurons of the olfactory epithelium but mainly in the migrating mass neurons (MM). Arrows indicated neurons migrating towards the telencephalon. **(M–P)** At HH21, *Nhlh1, Chga* and *Tagln3* were expressed in the neurons of the olfactory pit and in the migrating neurons **(M–O). (P)** *Cntn2* was only expressed in the migratory stream of neurons towards the telencephalon. Dotted lines highlight the olfactory pit. Scale bars: 100 μm.

In the chick olfactory system, neuroepithelium differentiation occurred as early as HH15 and is followed by migration of cells with a neuronal phenotype (HuC/D-positive) toward the telencephalic vesicles (De Carlos et al., 1995; Fornaro et al., 2003). In the present study, expression of *Nhlh1, Tagln3, Chga* and *Cntn2* was further analyzed in the olfactory placode at HH16 with HuC/D. *Nhlh1, Tagln3*, and *Chga* expression co-localized with a cluster of HuC/D-positive cells (Figures 5A–C), but *Cntn2* was not yet expressed (Figure 5D). At HH17, the placodal epithelium has thickened and was visible as an olfactory pit in the lateral position of the head. The number of HuC/D positive neurons significantly increased and were distributed throughout the epithelium but the density of *Nhlh1, Tagln3* and *Chga* expression was stronger in the lateral position at the level of the migratory cells (Figures 5E–G). This medial-superior position contained most of the differentiated neurons (Maier and Gunhaga, 2009). *Nhlh1, Tagln3* and* Chga*, expression was only detected in neurons migrating away from the placode into the mesenchyme, termed the migratory mass (MM; Figures 5E–G). This expression indicated that at HH17, differentiated neurons started to leave the olfactory placode and migrated away towards the olfactory bulb in the telencephalon.

At HH18, like at HH17, all the migratory cells that were *Nhlh1*-, *Tagln3*- and *Chga*-positive expressed HuC/D, however not all HuC/D cells expressed these genes (Figures 5I–K). *Cntn2* was also expressed in these HuC/D cells (Figure 5L). *Nhlh1, Talgn3* and *Cntn2* were not expressed in the migratory neurons at the tip of the migratory mass whereas *Chga* was expressed in all the neurons (Figures 5I–L; arrows). These results provide further evidence that differences in gene expression occurred during differentiation.

At HH21, the cells that migrated from the olfactory epithelium have generated a long bridge projecting toward the telencephalon (Drapkin and Silverman, 1999). Therefore, the nerve bundle was strongly immunoreactive for HuC/D and has a “fork shape” at the telencephalic exit point (Figures 5M–O). At this stage, there was little divergence in the expression pattern of *Nhlh1, Chga, Tagln3* and *Cntn2* (Figures 5M–P). *Nhlh1* and *Chga* expression was evenly distributed in both the olfactory epithelium and the migratory mass neurons, while *Cntn2* was expressed only in the migratory neurons (Figure 5P).

### Analysis in the developing spinal cord confirmed the expression of markers during different stages of neuronal differentiation

The spinal cord was used as a model system due to the highly organized and well-defined layers to better define the differential expression of these markers during different stages of neuronal differentiation. In this model, proliferating neural progenitor cells were located within the proliferative or ventricular zone (VZ), once differentiation starts the cells migrate through the intermediate zone (IZ) to the mantle zone (MZ; Leber and Sanes, 1995).

**Figure 6:**
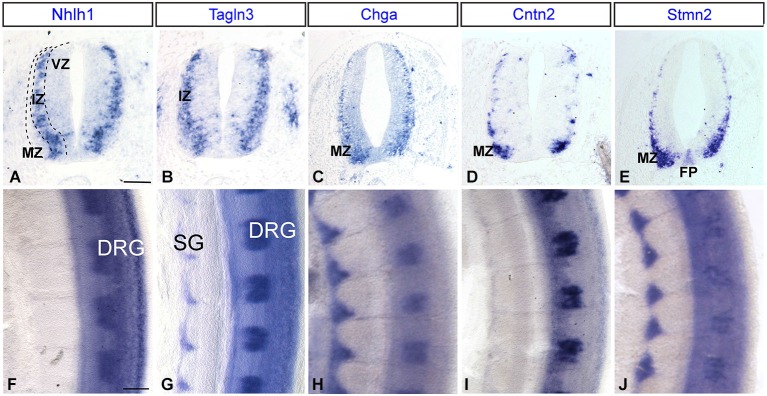
**Expression of *Nhlh1, Tagln3, Chga, Cntn2* and *Stmn2* by whole-mount *in situ* hybridization in the trunk spinal cord. (A–E)** Comparison of adjacent transverse sections of HH22 chick spinal cord. *Tagln3, Chga* and *Cntn2* expression was compared with *Nhlh1* and *Stmn2. Nhlh1* is an early marker expressed within the intermediate zone (IZ), *Tagln3* showed the same expression pattern. *Chga* and *Cntn2* displayed the same expression pattern as *Stmn2*, a known marker for mature neurons in the mantle zone (MZ). **(F–J)** Flat-mounted preparations of the spinal cord, all markers were expressed in the DRG. *Tagln3, Chga* and *Stmn2* were expressed in the SG. Scale bars: 100 μm. DRG: dorsal root ganglia; FP: floor plate; VZ: ventricular zone; SG: sympathetic ganglia.

The developmental expression pattern of *Tagln3, Chga* and *Cntn2* in the spinal cord at HH22 was compared with consecutive transverse sections, with *Nhlh1* and *Stmn2* (Figures 6A–E). In the IZ, the *Tagln3* expression domain overlapped considerably with expression of *Nhlh1* (Figures 6A,B). In comparison, *Chga* and *Cntn2* expression was restricted to the MZ, more laterally, like the late post-mitotic marker *Stmn2* (Figures 6C–E). A further difference, revealed *Stmn2* expression in the floor plate of the spinal cord, like in the rhombencephalon (Figure 3) that was not observed for the other markers (Figure 6E).

At HH22, *Tagln3, Chga* and *Stmn2* were expressed in the sensory neurons of the dorsal root ganglia (DRG) and the sympathetic ganglia (SG), whereas *Nhlh1* and *Cntn2* were only expressed in the DRG (Figures 6F–J). In the SG, *Chga* and *Stmn2* expression domains were larger than the *Tagln3* expression domain and extended dorsally encompassing the majority of the cells in the SG (Figures 6G–I). At this stage, most of the cells in the condensed SG have exited the cell cycle and the restriction of *Tagln3* expression to the ventral cells further supports the hypothesis that *Tagln3* was transiently activated during neurogenesis.

### The Notch response inhibitor DAPT enhanced expression of Tagln3, Chga and Cntn2 in the spinal cord

In a previous study, inhibition of Notch signaling during early development enhanced expression of several known neuronal markers such as *Nhlh1* and *Stmn2* as well as *Tagln3* and *Chga* in the developing hypothalamus (Ratié et al., 2013). In this current study, we have tested the expression of these genes at the level of the spinal cord when Notch signaling was lost through DAPT treatment.

**Figure 7:**
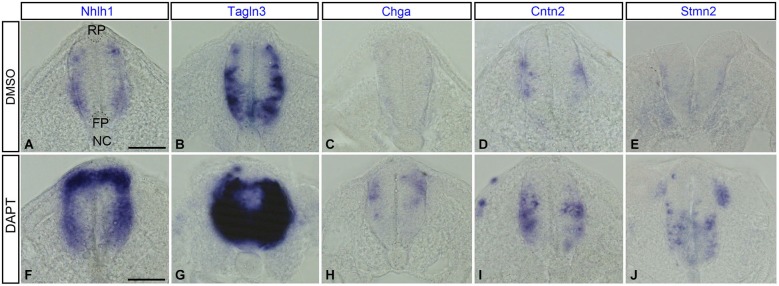
**Expression of *Nhlh1, Tagln3, Chga, Cntn2* and *Stmn2* in cross sections of the trunk spinal cord at HH14 after inhibition of Notch. (A–E)** Expression in DMSO-treated chick embryos (*n* = 12). **(F–J)** Expression in DAPT-treated chick embryos (*n* = 12). *Nhlh1, Tagln3, Chga, Cntn2* and *Stmn2* mRNA were overexpressed in DAPT treated spinal cord compared with DMSO treated spinal cord. Scale bars: 100 μm. FP: floor plate; NC: notochord; RP: roof plate.

As Notch signaling became established around HH9 in the spinal cord (Fior and Henrique, 2005), inhibition of the Notch pathway was performed from this stage. After an overnight culture with DMSO or the Notch inhibitor, DAPT, embryos were harvested at HH14 (*n* = 12 for each marker), when neurogenesis started in the chick neural tube (Le Dréau and Martí, 2012). Spinal cord sections revealed that *Nhlh1* and *Tagln3* were both upregulated in most of the cells throughout the spinal cord of DAPT-treated embryos when compared with DMSO-treated embryos with a complete penetrance (Figures 7A,B,F,G). This upregulation likely corresponded with an increase in the number of differentiating neurons within the IZ. Ectopic expression of *Chga, Cntn2* and *Stmn2* in the MZ of DAPT-treated embryos highlighted the upregulation even if the number of cells was much less than with *Nhlh1* and *Tagln3* (Figures 7C–E,H–J). *Chga* and *Stmn2* were weakly expressed along the pial surface of the neural tube in DMSO-treated embryos, but expression in DAPT-treated embryos also revealed precocious differentiation when Notch signaling was inhibited (Figures 7D,E,I,J).

## Discussion

In the present study, the spatial and temporal pattern of three Notch-dependent neuronal markers, *Tagln3, Chga* and *Cntn2*, were examined during early chick development together with the frequently used neuronal markers,* Nhlh1, Stmn2* and HuC/D (Groves et al., 1995; Murdoch et al., 1999; Fornaro et al., 2003; Ratié et al., 2013). The specific expression patterns of these markers in the nervous system provide us with clues into understanding the relationship between these genes and nerve development. This study also supports consistently the action that Notch signaling controls production of all neuronal progenitors.

### Expression throughout the developing chick nervous system

This study shows that *Tagln3, Chga* and *Cntn2* expression is confined to neurons suggesting that they are pan-neuronal markers, involved in broad differentiation of neuronal cells in both the central and peripheral nervous systems.

In the central nervous system, *Talgn3, Chga* and *Cntn2* are expressed in the early axon scaffold of the rostral brain, in columns of neurons at distinct dorso-ventral levels of the rhombencephalon and the spinal cord. In the latter, all markers are expressed in different zones of the neuroepithelium, leading to the hypothesis that these genes are required for different stages of differentiation. However, their expression domains in the neurons of the rostral brain suggest that they might also be involved in specific differentiation of distinct neuronal clusters (Wilson et al., 1990; Mastick and Easter, 1996; Ware and Schubert, 2011). The formation of these neuronal populations has to be tightly controlled as these tracts will act as a scaffold for later, follower axons. However, this process is not as well understood compared with other processes during caudal brain or spinal cord development, in part because of the lack of appropriate molecular markers (Guthrie, 2007; Briscoe and Novitch, 2008). This study reveals subtle differences of expression between *Tagln3, Chga* and *Cntn2* in these neuronal clusters. For example, *Chga* is the only marker expressed in the nTPC and *Cntn2* is absent in some populations where the other markers were expressed, such as the TPOC and the descending tract of the mesencephalic nucleus of the trigeminal nerve (DTmesV) neurons. The genes described in this study are new key markers and should be considered as active players in neural specificity.

In the peripheral nervous system,* Tagln3, Chga* and *Cntn2* genes are expressed in overlapping domains in the placodal derived components of the olfactory system, the cranial sensory ganglia as well as in the NCC derived components such as the DRG. This in agreement with a well characterized function of Notch in the NCC-deriving cells of the DRG (Hu et al., 2011). A major difference is *Tagln3, Chga* and *Cntn2* are expressed in a large number of cells of the cranial ganglia but only *Cntn2* is found in the migrating cells. Another exception was* Cntn2*, the only marker in this study to be expressed in the hypoglossal nerve (XII).

Despite the fact that the olfactory epithelium is a well-studied neurogenic territory in mouse as in chick (Cau et al., 2002; Maier and Gunhaga, 2009), we describe for the first time an early expression of *Nhlh1, Tagln3* and *Chga* in cells of the prospective olfactory placode at HH15 (equivalent to E9.5 in mouse; Fode et al., 1998) prior to invagination of the olfactory epithelium. The more restricted *Cntn2* expression domain in the migrating neurons compared to the broader *Nhlh1, Tagln3* and *Chga* expression, suggest that *Cntn2* is expressed in more mature olfactory neurons, while *Tagln3* and *Chga* are also expressed during the first steps of differentiation.

### Sequential expression during neuronal differentiation

The transition from a proliferative neural precursor cell to a post-mitotic neuron is a highly regulated process. Regulation of the Notch/proneural loop has been implicated in triggering a cascade of transcription factors such as *NeuroD* and *Nhlh1* (reviewed in Ishibashi, 2004; Louvi and Artavanis-Tsakonas, 2006). During neurogenesis, once a cell becomes post-mitotic, they migrate and concomitantly differentiate. Thus, an important aspect of studying neuronal cell differentiation is the identification of marker genes whose expression can be used to identify progressive stages in maturation of a cell during the differentiating program (Diez del Corral and Storey, 2001). Here, multiple evidence suggests that *Tagln3, Chga* and *Cntn2* are such chemical constituents involved in key steps of neurogenesis. *Nhlh1* and *Tagln3* appear to have a similar expression in all neuronal populations examined suggesting these genes are implicated in broad differentiation of neurons. This was particularly evident in the developing early axon scaffold neurons.

**Figure 8:**
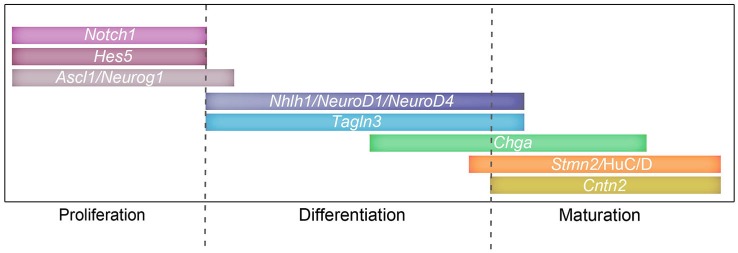
**Involvement of the Notch signaling pathway and target genes during proliferation and differentiation**. Schematic representing a possible timeline of expression for Notch components: *Notch1, Hes5*; proneural genes: *Ascl1, Neurog2, Neurog1*; and target genes: *NeuroD4, Nhlh1, Tagln3, Chga, Cntn2, Stmn2* and HuC/D during the different stages of neurogenesis.

Observations of the kinetic expression of these markers in direct comparison with HuC/D staining revealed that *Tagln3, Chga* and *Cntn2* are specifically expressed by post-mitotic neurons, but there are many subtle differences between them. For example, during olfactory neurogenesis (Figure 5), many *Tagln3*-, *Chga*- and *Cntn2*-positive cells expressed HuC/D, but not all HuC/D cells expressed these genes. Furthermore, some expression is also found in many HuC/D-negative cells. This result suggests either at these early stages there might be some subgroups of HuC/D expressing cells and/or that the differentiating program is not at the same step. Analysis of these markers in the spinal cord is especially enlightening because the neural maturation progression is easily visualized. Previous studies show an overlap of expression in the neural tube between *Tagln3* and* NeuroD4*, which identifies *Tagln3* as one of the earliest markers for post-mitotic neurons in the IZ of the chick spinal cord (Figure 8; Roztocil et al., 1997; Pape et al., 2008). In agreement with this, we observe that *Tagln3* expression coincides with that of *Nhlh1* in the IZ of the spinal cord but not with *Stmn2* in the lateral border of the MZ where mature neurons reside (Figure 8). To our knowledge, TAGLN3 appears to be the first structural protein associated to an early step of neural differentiation but not late maturation. This study concludes that *Nhlh1* and *Tagln3* expression is upregulated in differentiating cells that are not yet HuC/D positive while *Chga* and *Cntn2* are expressed in the later mature post-mitotic cells but not in all mature neurons throughout the nervous system (Figure 8). More generally, it appears that *Nhlh1* and *Tagln3* are expressed before the appearance of known neuronal markers such as Tuj1 and AChE, while *Chga* and *Cntn2* are later (Puelles et al., 1987; Ware and Schubert, 2011).

### Notch-dependent cascade of transcriptional regulators in neuronal development

While neuronal cells have various embryonic origins, the Notch/proneural gene feedback loop is the common feature for controlling their differentiation (Bertrand et al., 2002; Ishibashi, 2004; Louvi and Artavanis-Tsakonas, 2006). Studies in Rbpj−/− mutant mice have shown that the observed neurogenic phenotype is due to an increase of expression of the proneural genes, *Ascl1* and *Neurog* and the bHLH transcription factor *Nhlh1* leading to an excess of committed neuronal differentiation (de la Pompa et al., 1997). In DAPT-treated chick embryos, *Nhlh1* expression is similarly increased in the spinal cord and *Tagln3* overexpression is observed in the same region. While *Chga, Cntn2 and Stmn2* expression is increased in DAPT-treated embryos but at this early stage to a lesser extent further demonstrating that *Tagln3* is a marker of early neurogenesis, unlike *Chga* and *Cntn2*. These results show that an excess of committed neuronal precursors are generated which means eventually more mature neurons will be produced and therefore more *Chga* and *Cntn2* expression.

It is likely that Notch signaling plays a role in regulating the expression of these genes but further experiments will be required to determine if regulation of gene expression occurs directly by removal the HES repressive effect or through bHLH transcription factors as *Ascl1* or *Nhlh1*.

The sole requirement of the bHLH binding site, E-boxes located on many genes, can recapitulate the expression of *Cntn2* in the spinal cord (Hadas et al., 2013; Ratié et al., 2013). This suggests that bHLH proteins are the critical transcription factors regulating the expression of specific neuronal markers (Jacob et al., 2013) and it raises the possibility that *Tagln3, Chga and Cntn2* might represent a direct target gene of the transcription factor, *Nhlh1* (Hadas et al., 2013; Ratié et al., 2013).

### Evidence of evolutionary conservation of functions during vertebrate neurogenesis

Previous reports, although fractional, describe the expression of *Nhlh1, Tagln3, Chga, Cntn2* and* Stmn2* in vertebrates such as mouse, zebrafish and *Xenopus* (de la Pompa et al., 1997; Fode et al., 1998; Murdoch et al., 1999; Suzuki et al., 2003; Alves et al., 2010; Green and Vetter, 2011). Our observations in chick reveal that there is a considerable conservation of the expression domains of these genes with other vertebrates, suggesting there may also be an evolutionary conservation of function. For example, *Chga* expression in zebrafish is described in the developing cranial ganglia and other NCC-derived structures leading the authors to hypothesize that *Chga*-expressing cells are solely derived from NCCs (Xie et al., 2008). The *in situ* hybridization data presented here essentially does not differ from the pattern reported for the zebrafish but several other expression areas are described, in particular in non-NCC-derived. It suggests that *Chga* has a role during neurogenesis that is not specific to NCC-derived cells. In adults, CHGA has a key role in the formation of the neuroendocrine secretory system by interacting with STMN2 (Mahapatra et al., 2008). Our expression study suggests that the function of CHGA during neuronal differentiation may be based on the same interaction with STMN2.

*Cntn2* has commonly been used as a neuronal marker of post-mitotic neurons in the mouse spinal cord (Dodd et al., 1988) and extensive studies in zebrafish and mouse show a function for *Cntn2* during migration of facial branchiomotor neurons in the rhombencephalon (Garel et al., 2000; Sittaramane et al., 2009). This is in agreement with the strong expression of *Cntn2* observed in the same tissues in this study. *Cntn2* is also expressed in restricted subsets of central and peripheral neurons during adulthood and is implicated in the maintenance of the molecular organization of myelinated fibers (Denaxa et al., 2003; Karagogeos, 2003).

*Tagln3* expression has been described in the adult rat brain and chick embryonic nervous system (Depaz and Wilce, 2006; Pape et al., 2008), but no expression data is available in the mouse or zebrafish during early neurogenesis. Previously, a functional study in the chick embryo shows that an optimal level of TAGLN3 is required for neurite outgrowth of immature sensory neurons as well as of sympathetic neurons (Pape et al., 2008). In this current study, *Tagln3* expression is in nascent neurons that is consistent with a function that depends on actin cytoskeleton movements during early steps of neurogenesis and its sustained expression in postnatal brain implies a function in the maintenance of neuronal morphology (Depaz and Wilce, 2006).

## Concluding remarks

Collectively this data has been used to build a diagram representing a possible timeline of Notch (Hatakeyama and Kageyama, 2006; Kaltezioti et al., 2010), proneural genes and target genes (Roztocil et al., 1997; Maier and Gunhaga, 2009) during neurogenesis throughout the chick embryo (Figure 8). This study shows that several targets of the Notch/proneural network have interrelated expression patterns during chick neurogenesis, which suggest roles in neuronal class specification and differentiation. A better understanding of the function of these neuronal markers will enable us to further understand the complex formation of neurons.

## Author contributions

Leslie Ratié, Michelle Ware, Véronique David and Valérie Dupé set up and designed the experiments. Leslie Ratié, Michelle Ware and Hélène Jagline performed the experiments. Leslie Ratié, Michelle Ware and Valérie Dupé wrote the manuscript, and all authors have read, discussed and edited the manuscript.

## Conflict of interest statement

The authors declare that the research was conducted in the absence of any commercial or financial relationships that could be construed as a potential conflict of interest.

## Abbreviations

DAPT: N-[3.5-difluorophenacetyl-L-alanyl)]-S-phenylglycine t-butyl ester
DMSO: Dimethyl sulfoxide
DRG: dorsal root ganglia
DTmesV: descending tract of the mesencephalic nucleus of the trigeminal nerve
gV: trigeminal ganglion
gVI: facial ganglion
gVII: vestibulocochlear ganglion
gIX: petrosal ganglion
gX: nodose ganglion
IZ: intermediate zone
nIII: nucleus of oculomotor nerve
nIV: nucleus of trochlear nerve
NCC: neural crest cell
nMLF: nucleus of the medial longitudinal fascicle
nTPOC: nucleus of the tract of the postoptic commissure
MLF: medial longitudinal fascicle
nMTT: nucleus of mamillo-tegmental tract
MZ: mantle zone
OE: olfactory epithelium
r2: rhombomere 2
r4: rhombomere 4
SG: sympathetic ganglia
TPOC: tract of the postoptic commissure
VZ: ventricular zone.
